# Underreporting of nursing home utilization on the CMS-2728 in older incident dialysis patients and implications for assessing mortality risk

**DOI:** 10.1186/s12882-015-0021-9

**Published:** 2015-03-21

**Authors:** C Barrett Bowling, Rebecca Zhang, Harold Franch, Yijian Huang, Anna Mirk, William M McClellan, Theodore M Johnson, Nancy G Kutner

**Affiliations:** Birmingham/Atlanta Geriatric Research, Education, and Clinical Center, Atlanta Veterans Affairs Medical Center, 1670 Clairmont Road (11B), Decatur, GA 30033 USA; Division of General Medicine and Geriatrics, Department of Medicine, Emory University, Atlanta, GA USA; United States Renal Data System, Rehabilitation/Quality of Life Special Studies Center, Emory University, Atlanta, GA USA; Department of Biostatistics and Bioinformatics, Emory University, Atlanta, GA USA; Division of Renal Medicine, Department of Medicine, Emory University, Atlanta, GA USA; Subspecialty Service Line, Atlanta Veterans Affairs Medical Center, Decatur, GA USA; Departments of Medicine and Epidemiology, Emory University, Atlanta, GA USA

**Keywords:** Nursing home, End-stage renal disease, Frail elderly

## Abstract

**Background:**

The usage of nursing home (NH) services is a marker of frailty among older adults. Although the Centers for Medicare & Medicaid Services (CMS) revised the Medical Evidence Report Form CMS-2728 in 2005 to include data collection on NH institutionalization, the validity of this item has not been reported.

**Methods:**

There were 27,913 patients ≥ 75 years of age with incident end-stage renal disease (ESRD) in 2006, which constituted our analysis cohort. We determined the accuracy of the CMS-2728 using a matched cohort that included the CMS Minimum Data Set (MDS) 2.0, often employed as a “gold standard” metric for identifying patients receiving NH care. We calculated sensitivity, specificity, positive predictive value (PPV), and negative predictive value (NPV) for the CMS-2728 NH item. Next, we compared characteristics and mortality risk by CMS-2728 and MDS NH status agreement.

**Results:**

The sensitivity, specificity, PPV and NPV of the CMS-2728 for NH status were 33%, 97%, 80% and 79%, respectively. Compared to those without the MDS or CMS-2728 NH indicator (No MDS/No 2728), multivariable adjusted hazard ratios (95% confidence interval) for mortality associated with NH status were 1.55 (1.46 – 1.64) for MDS/2728, 1.48 (1.42 – 1.54) for MDS/No 2728, and 1.38 (1.25 – 1.52) for No MDS/2728. NH utilization was more strongly associated with mortality than other CMS-2728 items in the model.

**Conclusions:**

The CMS-2728 underestimated NH utilization among older adults with incident ESRD. The potential for misclassification may have important ramifications for assessing prognosis, developing advanced care plans and providing coordinated care.

**Electronic supplementary material:**

The online version of this article (doi:10.1186/s12882-015-0021-9) contains supplementary material, which is available to authorized users.

## Background

Older adults are the fastest growing group initiating dialysis in the United States [1,2]. In 2011, for persons 75 years and older the adjusted incidence rate of end-stage renal disease (ESRD) was 1,707 per million population, up 7.1% from 2000 [1]. For older adults, progression to ESRD carries a poor prognosis and increased risk for hospitalization and death [3-5]. Although frailty and functional impairment have been shown to be common and associated with poor health outcomes in ESRD, these measures are not commonly captured in the routine care of these patients [4,6,7].

The usage of nursing home (NH) services is a marker of frailty among older adults [8]. Prior studies have shown that dialysis initiation may precede loss of independence and need for nursing home placement [3]. Additionally, among long-term NH residents dialysis initiation was shown to be associated with persistent functional decline and reduced survival [5]. However, data on NH care among incident ESRD patients is limited [9,10]. Failing to recognize NH utilization as a significant risk factor for mortality may impact prognostication and clinical decision making for older patients with advanced kidney disease.

In 2005 the Centers for Medicare & Medicaid Services (CMS) Medical Evidence Report (Form CMS-2728) was revised to include data collection on whether or not the patient is institutionalized in a NH. While this new data collection provides the opportunity to describe the utilization and outcomes associated with institutional care, the validity of the CMS-2728 to identify NH patients has not been reported. Therefore, the aims of the current analysis were to report the accuracy of the CMS-2728 NH item among incident ESRD patients ≥ 75 years old using a matched cohort that included the CMS Long Term Care Minimum Data Set (MDS), a “gold standard” metric for identifying patients receiving NH care. In order to assess potential implications for failing to recognize or report NH utilization, we compared mortality risk by CMS-2728 and MDS NH status agreement.

## Methods

### Data sources and participants

Data were obtained through the United States Renal Data System (USRDS). The current analysis was limited to 27,913 patients ≥ 75 years old with incident ESRD and completion of the CMS-2728 during 2006. We chose this age cut point because of the increase in proportion of US adults requiring nursing home services after the age of 75 years compared to ≤ 74 years of age [11]. Data on other covariates were obtained through the CMS-2728 and includes the demographic factors of age, gender, race, ethnicity, Medicaid coverage, region of residence (defined by US Census Regions Northeast, South, Midwest and West). Treating physicians are required to complete the CMS-2728 form within 45 days of the first maintenance renal replacement therapy for all ESRD patients regardless of current or future intention to use Medicare [12]. Health characteristics included primary cause of renal failure, cardiovascular disease (CVD) comorbidity, functional impairment (inability to ambulate, inability to transfer or assistance needed with activities of daily living), and laboratory values included serum albumin, serum creatinine and hemoglobin. Treatment characteristics included primary dialysis setting, primary type of dialysis, receipt of predialysis nephrology care and initial type of hemodialysis vascular access. Item 17u is included in the section to be completed by the attending physician and asks if the patient is “Institutionalized.” Patients that are institutionalized are further characterized as “1. Assisted living,” “2. Nursing Home,” or “3. Other Institution.” To allow for a complete year of data collection with the newly introduced institutionalization item on the CMS-2728, our analysis was limited to 2006. This study protocol was approved by the Emory University Institutional Review Board. Written informed consent was not obtained.

### Nursing home status agreement

In order to determine the accuracy of the CMS-2728 we used a matched cohort that included the CMS Long Term Care MDS 2.0 for 2006 obtained from the USRDS [13]. The MDS is a standardized, primary screening and assessment tool of health status for all residents admitted to a Medicare or Medicaid certified which includes 95% of all US NHs [14]. CMS collects MDS data through surveys that are administered on admission, quarterly, annually, or after a significant change in status (e.g., discharge from NH). Patients with MDS data were matched with the USRDS database by USRDS ID.

For our primary analysis, patients were defined as receiving NH care by the MDS 1) if the last MDS record prior to the ESRD date indicated that they were in a NH or 2) if they had an MDS record indicating admission to a NH within 30 days after the ESRD date. Prior studies have shown delays in completion of the CMS-2728, [15] therefore we chose a window of 30 days after the ESRD date in order to capture those most likely to be receiving NH care at time of or immediately following dialysis initiation. A 30 day window was chosen based on the Medicare post-acute care benefit that allows for skilled nursing care in a nursing home following an acute hospitalization. In secondary analyses, three additional definitions were considered including 1) an MDS record prior to the ESRD date without the 30 day window following the ESRD date, 2) using the CMS-2728 physician signature date instead of the ESRD date, and 3) using any institutionalization on CMS-2728 item 17u (“1. Assisted living,” “2. Nursing Home,” or “3. Other Institution”).

### Mortality

Mortality subsequent to the ESRD date was determined using data from the USRDS that includes the CMS ESRD Death Notification (CMS-2746) and social security death index. A death notification is required when ESRD patients die, regardless of payer status [12]. Follow-up for the current analysis was available through 2012.

### Statistical analyses

We examined the cross tabulations of NH status ascertained by MDS and by the CMS-2728 and calculated the sensitivity, specificity, positive predictive value (PPV), and negative predictive value (NPV) for the CMS-2728 item 17u. Additionally, we calculated the mean length of NH stay in days from the MDS for those identified by both MDS and the CMS-2728 versus those identified only by MDS during the window of 100 days before and 130 days after the ESRD date. This time window was chosen to capture ESRD patients requiring NH stays that exceed the 100 days of skilled nursing facility care that is covered by Medicare Part A after a qualifying hospital stay. Demographic, health and treatment characteristics were calculated as proportions or means and standard deviation as appropriate by 2728 and MDS NH status agreement category (i.e., MDS/2728, MDS/No 2728, No MDS/2728, No MDS/No 2728). To assess for patient characteristics associated with having the CMS-2728 indicator among those identified as receiving NH care by MDS (i.e., sensitivity), we calculated crude and multivariable adjusted odds ratios of demographic, health and treatment characteristics with the CMS-2728 NH status using logistic regression limited to participants in a NH by the MDS. Next we calculated crude and multivariable adjusted odds ratios of demographic, health and treatment characteristics associated with not having the CMS-2728 NH indicator limited to participants without an MDS record (i.e., specificity). Multivariable adjustment included age, sex, ethnicity, race, Medicaid use, region of residence, cause of renal failure, CVD comorbidity, functional impairment, serum albumin, serum creatinine, hemoglobin, primary dialysis setting, predialysis nephrology care, initial type of hemodialysis vascular access and incident ESRD year. Patients were categorized as having functional impairment if they had at least one of the following: inability to ambulate, inability to transfer or needs assistance with daily activities. Finally, using the Kaplan-Meier method we estimated the cumulative mortality by CMS-2728 and MDS NH status agreement category. Associations of NH status defined by 1) MDS alone, 2) CMS-2728 alone and 3) MDS/2728 agreement (MDS/2728, MDS/No 2728, No MDS/2728, No MDS/No 2728) with all-cause mortality were examined by calculating mortality rates and hazard ratios using three separate Cox proportional hazards models. For the model with NH status agreement categories, those with No MDS/No 2728 served as the referent group. Multivariable adjustment included all demographic, health and treatment factors described above. SAS, version 9.2 (SAS Institute, Cary NC) was used for all analyses.

## Results

### Nursing home status reporting on the CMS-2728

Of the 7,801 (27.9%) dialysis patients for whom the MDS indicated that they were in a NH prior to or 30 days after the ESRD date, 2,565 (33%) were identified by the CMS-2728. In contrast, of the 20,112 participants without MDS records for NH care, 19,456 (97%) were not in a NH according to the CMS-2728. The PPV and NPV of the CMS-2728 for NH status were 80% and 79%, respectively (Table 1). Among those for whom the MDS indicated that they were in a NH, the mean (standard deviation) number of NH days was 72.5 (56.0) for those identified by CMS-2728 versus 48.3 (47.3) among those not identified as receiving NH care (p <0.001). The accuracy of the CMS-2728 NH item when different definitions were considered is displayed in Additional file 1 Table S1.

**Table 1:** **Accuracy of the Centers for Medicare & Medicaid Services (CMS) Medical Evidence Report Form CMS-2728 nursing home status versus the “gold standard” CMS nursing home Minimum Data Set (MDS) among incident dialysis patients ≥ 75 years (n = 27,913)**

	**MDS (n)**		**Sensitivity**	**Specificity**	**PPV**	**NPV**
**CMS-2728 (n)**	**Yes**	**No**				
**Yes**	2,565	656	0.33	0.97	0.80	0.79
**No**	5,236	19,456	(0.32, 0.34)	(0.96, 0.97)	(0.78, 0.81)	(0.78, 0.79)

PPV = positive predictive value.

NPV = negative predictive value.

Nursing home status for the CMS-2728 defined as “2. nursing home” on item 17u.

Nursing home status for the MDS was defined by an MDS record prior to the ESRD date indicating that the patient was in a nursing home or an MDS record indicating admission to a nursing home within 30 days after the ESRD date.

### ESRD patient characteristics and nursing home status

Demographic, health and treatment characteristics for ESRD patients ≥ 75 years old by CMS-2728 and MDS NH status agreement category are displayed in Table 2. Overall, patients were similar in age by NH status category. Among patients for whom the MDS indicated that they were in a NH, those with the CMS-2728 NH indicator were more likely to be women, to be black, have Medicaid coverage and live in the South or Midwest compared to the Northeast or West. Additionally, within this group, those with the CMS-2728 NH indicator were much more likely to have an inability to ambulate (41.4% versus 8.0%), inability to transfer (25.3% versus 3.3%) or need assistance with daily activities (56.3% versus 11.7%). However, those without MDS data, but with the CMS-2728 NH indicator (i.e., No MDS/2728) had the highest reported prevalence of functional impairment on the CMS-2728.

**Table 2:** **Demographic, health and treatment characteristics of incident dialysis patients ≥ 75 years old by CMS-2728 and MDS nursing home status agreement (n = 27,913)**

	**MDS (n = 7,801)**		**No MDS (n = 20,112)**	
	**2728**	**No 2728**	**2728**	**No 2728**
	**(n = 2,565**)	**(n = 5,236)**	**(n = 656**)	**(n = 19,456)**
**Demographic characteristics**				
Age, mean (SD)	81.7 (4.7)	81.6 (4.6)	81.0 (4.5)	80.5 (4.3)
Women, (%)	55.3	51.1	50.3	43.3
Hispanic or Latino	5.8	5.7	7.0	9.7
Race				
White	76.8	81.4	77.0	78.5
Black	20.8	15.3	19.5	16.9
American Indian/Alaskan	0.1	0.4	0.3	0.4
Asian	1.8	2.4	2.9	3.3
Other	0.5	0.5	0.3	0.9
Medicaid	28.8	17.5	23.8	15.1
Region of residence				
Northeast	21.9	28.7	24.2	20.2
South	35.2	28.6	35.7	36.3
Midwest	31.4	25.0	25.9	22.9
West	11.6	17.6	14.3	20.6
**Health characteristics**				
Primary cause of renal failure				
Diabetes	39.2	36.0	39.5	34.4
Glomerular nephritis	3.0	3.8	2.1	4.7
Secondary glomerulonephritis /vasculitis	0.7	1.0	0.9	0.9
Interstitial nephritis	3.9	3.0	2.6	3.7
Hypertension/large vessel	37.3	40.2	35.8	42.1
Cystic/hereditary/congenital	0.4	0.5	0.5	1.5
Neoplasms/tumors	1.8	2.2	1.7	3.0
Other	13.8	13.4	16.9	9.6
CVD Comorbidity				
Congestive heart failure	53.3	52.4	54.1	41.9
Atherosclerotic heart disease	32.6	35.0	35.4	32.7
Cerebrovascular disease	19.7	14.4	21.7	11.0
Peripheral vascular disease	21.1	20.2	27.3	18.3
Other	30.0	23.2	35.2	21.3
Functional impairment				
Inability to ambulate	41.4	8.0	51.5	5.2
Inability to transfer	25.3	3.3	34.2	2.2
Needs assistance with daily activities	56.3	11.7	58.8	11.3
Serum albumin, g/dL, mean (SD)	2.9 (0.6)	3.0 (0.6)	2.8 (0.7)	3.3 (0.7)
Serum creatinine, mg/dL, mean (SD)	5.1 (2.3)	5.2 (2.4)	5.0 (2.3)	5.5 (2.4)
Hemoglobin, g/dL, mean (SD)	10.2 (1.6)	10.2 (1.6)	10.1 (1.6)	10.4 (1.6)
**Treatment characteristics**				
Primary dialysis setting				
Home	1.6	0.8	1.5	4.8
Dialysis facility/Center	94.1	98.0	92.5	95.1
Skilled nursing facility/Long term care facility	4.3	1.2	6.0	0.1
Primary type of dialysis				
Hemodialysis	99.1	99.4	99.4	95.4
CAPD	0.7	0.4	0.2	3.1
CCPD	0.2	0.2	0.5	1.5
Other	0.0	0.0	0.0	0.0
Predialysis nephrology care	41.4	45.4	34.6	64.7
Initial type of hemodialysis access				
AVF	4.2	5.1	3.5	16.0
Graft	3.3	2.7	3.1	5.3
Catheter	91.4	90.7	91.9	77.4
Other	1.2	1.6	1.5	1.3
CVD = cardiovascular disease				

All numbers are percentage or mean (SD).

Among the 7,801 incident ESRD patients ≥ 75 years old identified by the MDS as receiving NH care, having Medicaid coverage, residing in the South or Midwest, and having diabetes as the primary cause of ESRD, were associated with a higher odds of being correctly identified as receiving NH care on the CMS-2728 (Table 3). The multivariable adjusted odds ratio (95% confidence interval [CI]) for the association of functional impairment with the CMS-2728 NH indicator was 10.42 (9.10 – 11.93). Characteristics associated with not having a CMS-2728 NH indicator among those without an MDS record (i.e., modeling specificity), included Hispanic ethnicity, serum albumin, and predialysis care. Residing in the Northeast, cerebrovascular disease, peripheral vascular disease, functional impairment and having an arteriovenous fistula were associated with a lower odds of being correctly identified as not receiving NH care.

**Table 3:** **Crude and multivariable adjusted odds ratios (95% confidence intervals) for sensitivity and specificity of CMS-2728 nursing home indicator among incident dialysis patients ≥ 75 years**

	**Sensitivity (n = 7,801)**		**Specificity (n = 20,112)**	
	**Crude**	**Multivariable adjusted**	**Crude**	**Multivariable adjusted**
**Age, years**				
**75 – 79**	1.00	1.00	1.00	1.00
**80 – 84**	1.01 (0.90, 1.13)	1.08 (0.92, 1.26)	0.81 (0.68, 0.96)	0.80 (0.64, 1.01)
**85 – 89**	0.98 (0.86, 1.12)	1.04 (0.86, 1.25)	0.71 (0.57, 0.88)	0.77 (0.58, 1.02)
≥**90**	1.24 (1.01, 1.51)	1.22 (0.92, 1.62)	0.86 (0.55, 1.34)	0.81 (0.48, 1.38)
**Women (vs. men)**	1.18 (1.08, 1.30)	1.07 (0.94, 1.23)	0.76 (0.65, 0.88)	1.06 (0.86, 1.31)
**Hispanic or Latino (vs. non-Hispanic)**	1.01 (0.83, 1.24)	0.90 (0.67, 1.21)	1.42 (1.05, 1.92)	2.01 (1.31, 3.06)
**Race**				
**White**	1.00	1.00	1.00	1.00
**Black/African American**	1.44 (1.28, 1.63)	1.08 (0.90, 1.30)	0.85 (0.70, 1.04)	1.29 (0.97, 1.72)
**American Indian/Alaska**	0.28 (0.09, 0.94)	0.16 (0.03, 0.81)	1.14 (0.28, 4.67)	1.40 (0.18, 10.90)
**Native Asian**	0.79 (0.56, 1.11)	1.27 (0.80, 2.03)	1.12 (0.71, 1.79)	1.17 (0.66, 2.07)
Other*	1.08 (0.54, 2.17)	0.80 (0.29, 2.21)	3.02 (0.75, 12.2)	--
**Medicaid (vs. no Medicaid)**	1.91 (1.70, 2.13)	1.88 (1.59, 2.22)	0.57 (0.47, 0.69)	0.76 (0.58, 0.99)
**Region of residence**				
**Northeast**	1.16 (0.98, 1.36)	1.12 (0.89, 1.40)	0.58 (0.45, 0.76)	0.72 (0.52, 1.00)
**South**	1.87 (1.60, 2.18)	1.55 (1.24, 1.94)	0.71 (0.56, 0.90)	1.07 (0.78, 1.46)
**Midwest**	1.90 (1.63, 2.23)	1.96 (1.56, 2.46)	0.62 (0.48, 0.80)	0.88 (0.63, 1.24)
**West**	1.00	1.00	1.00	1.00
**Primary cause of ESRD (diabetes vs. other)**	1.15 (1.04, 1.26)	1.16 (1.00, 1.33)	0.81 (0.69, 0.94)	0.81 (0.65, 1.01)
**CVD Comorbidity (vs. no CVD comorbidity)**				
**Congestive heart failure**	1.04 (0.84, 1.14)	0.89 (0.77, 1.02)	0.61 (0.52, 0.72)	0.99 (0.80, 1.23)
**Atherosclerotic heart disease**	0.90 (0.81, 0.99)	0.88 (0.76, 1.03)	0.89 (0.75, 1.04)	1.25 (0.99, 1.58)
**Cerebrovascular disease**	1.45 (1.28, 1.65)	1.14 (0.96, 1.36)	0.45 (0.37, 0.54)	0.75 (0.58, 0.96)
**Peripheral vascular disease**	1.06 (0.94, 1.19)	0.87 (0.73, 1.03)	0.60 (0.50, 0.71)	0.69 (0.55, 0.88)
**Other**	1.41 (1.27, 1.57)	1.15 (0.99, 1.33)	0.50 (0.42, 0.59)	0.80 (0.64, 0.99)
Functional impairment (vs. no functional impairment)**	11.21 (10.02, 12.53)	10.42 (9.10, 11.93)	0.06 (0.05, 0.08)	0.09 (0.07, 0.11)
**Serum albumin (per 1 g/dL)**	0.87 (0.80, 0.94)	0.96 (0.86, 1.07)	2.66 (2.34, 3.02)	1.80 (1.54, 2.10)
**Serum creatinine (per 1 mg/dL)**	0.98 (0.96, 1.00)	1.00 (0.97, 1.02)	1.13 (1.08, 1.17)	1.07 (1.01, 1.12)
**Hemoglobin (per 1 g/dL)**	0.99 (0.96, 1.03)	1.00 (0.96, 1.05)	1.14 (1.08, 1.20)	1.01 (0.94, 1.08)
Dialysis facility/Center (vs. SNF)***	0.28 (0.21, 0.38)	0.51 (0.33, 0.81)	--	--
**Predialysis nephrology care (vs. No care)**	0.83 (0.75, 0.92)	0.86 (0.75, 1.00)	3.19 (2.67, 3.82)	2.04 (1.62, 2.57)
**Arteriorvenous fistula (vs. Graft)**	0.68 (0.47, 0.96)	0.80 (0.50, 1.28)	2.61 (1.43, 4.77)	1.83 (0.90, 3.76)

CVD = cardiovascular disease, SNF = skilled nursing facility.

*unstable estimates due to very small numbers in the other race category includes one of more of inability to ambulate.

**inability to ambulate, transfer, or needs assistance with daily activities.

***unstable estimates due to very small numbers receiving dialysis in SNF.

Multivariable adjustment includes age, sex, ethnicity, race, Medicaid coverage, region of residence, primary cause of dialysis, CVD comorbidity, functional impairment, serum albumin, serum creatinine, hemoglobin, primary dialysis setting, predialysis nephrology care, initial type of dialysis access and incident ESRD year.

### Nursing home status and mortality

**Figure 1:**
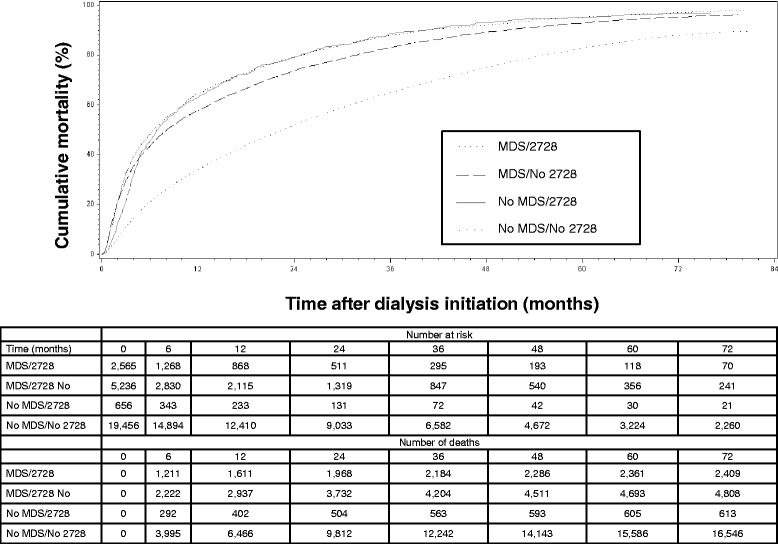
**Survival curves (Kaplan-Meier method) for incident ESRD patients** ≥ **75 years old by CMS-2728 and Minimum Data Set (MDS) nursing home status agreement.**

Overall, there were 24,642 (88%) deaths over a median 426 days of follow-up. Mortality rates per 1,000 person years were 802, 656, 770 and 357 by NH status agreement categories MDS/2728, MDS/No 2728, No MDS/2728, No MDS/No 2728, respectively. Patients identified by either the MDS or CMS-2728 had a greater risk for death over follow-up compared to those who were not identified has receiving NH care by the MDS or CMS-2728 (Figure 1). Compared to those without the MDS or CMS-2728 NH indicator (No MDS/No 2728), multivariable adjusted hazard ratios (95% CI) for mortality associated with NH status were 1.55 (1.46–1.64) for MDS/2728, 1.48 (1.42–1.54) for MDS/No 2728, and 1.38 (1.25 – 1.52) for No MDS/2728 (Table 4). When modelled separately, both NH status defined by MDS alone or CMS-2728 alone were independently associated with mortality (top panel Table 4).

**Table 4:** **Crude and multivariable adjusted hazards ratios (95% confidence intervals) for mortality among incident dialysis patients ≥ 75 years old (n = 27,913)**

	**Crude**	**Multivariable adjusted**
NH Status – by MDS*		
No MDS (n = 20,112)	**1 (ref)**	**1 (ref)**
MDS (n = 7,801)	**1.81 (1.76, 1.86)**	**1.47 (1.42, 1.52)**
NH Status – by CMS-2728 **		
No 2728 (n = 24,692)	**1 (ref)**	**1 (ref)**
2728 (n = 3,221)	**1.85 (1.78, 1.92)**	**1.35 (1.28, 1.42)**
NH status agreement***		
MDS/2728	2.08 (1.99, 2.17)	1.55 (1.46, 1.64)
MDS/No 2728	1.75 (1.69, 1.81)	1.48 (1.42, 1.54)
No MDS/2728	1.97 (1.82, 2.13)	1.38 (1.25, 1.52)
No MDS/No 2728	1 (ref)	1(ref)
**Age, years**		
75 – 79	1 (ref)	1 (ref)
80 – 84	1.23 (1.19, 1.26)	1.17 (1.13, 1.21)
85 – 89	1.51 (1.46, 1.57)	1.40 (1.34, 1.46)
≥90	1.93 (1.81, 2.06)	1.69 (1.57, 1.82)
Women	0.96 (0.93, 0.98)	0.87 (0.85, 0.90)
Hispanic or Latino	0.85 (0.81, 0.89)	0.75 (0.70, 0.79)
Race		
White	1 (ref)	1 (ref)
Black or African American	0.79 (0.76, 0.81)	0.77 (0.74, 0.81)
American Indian/Alaska Native	0.82 (0.67, 1.02)	0.89 (0.70, 1.14)
Asian	0.71 (0.66, 0.77)	0.77 (0.70, 0.84)
Other	0.72 (0.62, 0.83)	0.65 (0.53, 0.79)
Medicaid	1.01 (0.97, 1.04)	1.03 (0.99, 1.08)
Region of residence		
Northeast	1.10 (1.06, 1.14)	0.97 (0.92, 1.01)
South	1.15 (1.11, 1.19)	1.09 (1.04, 1.14)
Midwest	1.11 (1.07, 1.15)	0.95 (0.90, 0.99)
West	1 (ref)	1 (ref)
Primary cause of ESRD (diabetes vs. other)	1.01 (0.98, 1.03)	1.02 (0.99, 1.06)
CVD Comorbidity		
Congestive heart failure	1.37 (1.34, 1.41)	1.18 (1.14, 1.22)
Atherosclerotic heart disease	1.16 (1.13, 1.19)	1.03 (0.99, 1.06)
Cerebrovascular disease	1.22 (1.17, 1.26)	1.06 (1.01, 1.11)
Peripheral vascular disease	1.21 (1.17, 1.24)	1.08 (1.03, 1.12)
Other	1.26 (1.22, 1.29)	1.09 (1.06, 1.13)
Functional impairment†	1.60 (1.55, 1.65)	1.25 (1.20, 1.31)
Serum albumin (g/dL)	0.75 (0.73, 0.76)	0.85 (0.83, 0.87)
Serum creatinine (mg/dL)	0.95 (0.95, 0.96)	0.97 (0.96, 0.97)
Hemoglobin (g/dL)	0.97 (0.97, 0.98)	0.99 (0.98, 1.00)
Dialysis facility/Center (vs. SNF)	0.65 (0.57, 0.74)	1.17 (0.99, 1.38)
Predialysis nephrology care (vs. No care)	0.74 (0.72, 0.76)	0.90 (0.87, 0.94)
Arteriorvenous fistula (vs. Graft)	0.92 (0.86, 0.99)	0.91 (0.84, 0.99)

CVD = cardiovascular disease, SNF = skilled nursing facility.

†includes one of more of inability to ambulate, inability to transfer, or needs assistance with daily activities.

*Model 1 – NH status defined by MDS alone.

**Model 2 – NH status defined by CMS-2728 alone.

***Model 3 – NH status defined by MDS/2728 agreement category.

For each model multivariable adjustment includes age, sex, ethnicity, race, Medicaid coverage, region of residence, primary cause of dialysis, CVD comorbidity, functional impairment, serum albumin, serum creatinine, hemoglobin, primary dialysis setting, predialysis nephrology care, initial type of dialysis access and incident ESRD year.

## Discussion

For incident ESRD patients ≥ 75 years old, the CMS-2728 may underestimate the true utilization of NH care in this population, however those identified by the CMS-2728 as being in a NH have a high probability of actually being in a NH (i.e., high PPV). When analyses were repeated with different definitions for NH status, the sensitivity remained low. Additionally, we found that NH utilization defined by either the MDS or CMS-2728 was associated with mortality. Because other markers of frailty may not be routinely collected in this population, inaccurate reporting on the CMS-2728 NH item may mask important clinical information about older adults with incident ESRD. Our findings suggest a gap in recognition of NH care that may have important implications for assessing prognosis, advanced care planning and providing coordinated care.

Consistent with prior studies, we found that incident ESRD carries a poor prognosis for older adults [4,5]. Overall, 88% of incident ESRD patients ≥ 75 years old died during follow-up. Compared to those with neither the MDS or CMS-2728 NH indicator (No MDS/No 2728), presence of either NH indicator was associated with increased mortality. For patients identified by either the MDS or CMS-2728 as receiving NH care, the first 6 months was a high risk time with 47-50% of all deaths for these groups occurring during this time period. In contrast, among those not identified as receiving NH care by either the MDS or CMS-2728 (n = 19,456), only 24% of the total deaths for this group occurred in the first 6 months. Similar mortality risk between the MDS/2728 and MDS/No 2728 groups suggests that relying on the Medical Evidence Report form alone leads to misclassification of a large proportion of older incident ESRD patients who have a poor prognosis. Reinforcing the need for accurate reporting of NH status are prior studies showing that comorbidities alone may not be as helpful for risk prediction in older adults. For example, Cheung and colleagues recently reported the poor performance of prognostic indices in ESRD that rely primarily on medical comorbidities [16]. In the current study, we found that NH utilization was more strongly associated with mortality risk than all other CMS-2728 items included in our model and the magnitude of this association was on the order of a ten year increase in age. Indices that take into account not just comorbidities, but contextual factors such as markers of frailty, the need for NH care, or healthcare intensity at the time of dialysis initiation may have the potential to improve prognostication [17,18].

In the current analysis, the CMS-2728 underreported the proportion of incident ESRD patients ≥ 75 years old receiving NH care by two thirds. Similarly, a low sensitivity for the identification of patient characteristics such as smoking status or reported predialysis nephrology care has been previously described [19-21]. These discrepancies have been speculated to be due to nephrologists not having access to the context of the whole patient as may occur during urgent dialysis initiation, completion of the form by dialysis unit personnel who may not be familiar with the patient’s full medical history or ambiguity in the CMS-2728 form itself [19,22]. Along these lines, there are several potential explanations for the low sensitivity of the CMS-2728 NH status. First, providers completing the CMS-2728 may not be aware if the patient is currently receiving NH care or may not believe that this is important information to accurately report. The patient may not have been asked or in the case of delirium or critical illness the patient might not be able to say. Additionally, providers may not be familiar with language used on CMS-2728 or the time frame over which to consider NH status. The term “institutionalized” may be interpreted as requiring long term care rather than post-acute care in a skilled nursing facility care that is covered by Medicare Part A after a qualifying hospital stay. This may be supported by our finding of a higher average number of NH days among those identified by the CMS-2728 compared to those not identified as receiving NH care. Differences between assisted living, NH and other institution may not be clear especially as patients may be transitioning from one level of care to another, [23] however including assisted living and other institution in a secondary analysis only resulted in a small increase in the sensitivity. Lastly, because of the number of transitions between care sites near the time of dialysis initiation, patients may have recently transitioned out of or into a NH. In this case, the provider may be aware of the patient’s NH status, but neither the CMS-2728, nor the MDS, may provide the granularity necessary to determine if a patient was in a NH on the exact ESRD date. One approach to improving the performance of the CMS-2728 to identify patients receiving NH and other markers of frailty is to remove these from the comorbidities section (item 17) and creating a separate section on frailty, function and nursing home care using standardized and previously validated assessments.

Despite the low sensitivity, 80% of those identified by the CMS-2728 had MDS data indicating that they were receiving NH care at the time of dialysis initiation. These findings have important research implications. For outcomes research purposes where the aim is to create a cohort with a known condition using CMS data such as the CMS-2728, having a high PPV is important to ensure the majority of patients have the condition of interest [24]. Using Medicare claims alone to develop an analytic cohort of patients receiving NH care can be quite challenging [25]. In fact, compared to previous studies using complex Medicare claims algorithms to identify patients receiving NH care, we found a similar PPV using only the single Item 17u on the CMS-2728 [13]. Additionally, we found a similarly high NPV. Having both a high PPV and NPV has been described as being important for comparing those with versus without a certain condition. Our findings support the use of the CMS-2728 NH indicator to define NH status as has been recently used in a sub-group analysis of dialysis timing and outcomes in older adults [26]. In the current study, we identified cross sectional associations between NH status and functional impairment and a prospective association with mortality risk that was robust to multivariable adjustment for several mortality risk factors corresponding to a high predictive validity of the CMS-2728 NH item.

Educational resources exist to identify and address issues related to overall health status such as frailty and functional decline in order to improve outcomes [27,28]. However, currently there are no specific recommendations for older ESRD patients requiring NH care. Findings from the current study suggest possible gaps in the coordination of care between dialysis units and nursing facilities and an opportunity to improve recognition and reporting of NH care among incident ESRD patients ≥ 75 years of age. In addition, despite a considerably lower proportion of reported functional impairment among those missed by the CMS-2728, mortality risk was similar to those identified by both the MDS and CMS-2728 as receiving NH care. Future studies may be necessary to determine whether or not NH care itself contributes to the poor outcomes in this group or is merely a marker of disease burden and declining health status. Regardless, recognizing the mortality risk associated with the need for NH care, even among patients without reported functional impairment, may provide important information to support clinical decision making and communication with patients about advance care planning.

Potential limitations include the reliance on data from the CMS-2728 to identify patient demographic, medical and treatment characteristics. Prior studies have shown substantial disagreement between the CMS-2728 and Medicare claims for certain items including predialysis nephrology care [15,16]. Additionally, lack of instructions or clarification on the NH item with regards to terminology (i.e., institutionalized, assisted living, NH, other institution) may lead to misclassification. Institutionalization is listed under co-morbid conditions and the instructions state to “check all that apply currently and/or during the last 10 years” however, MDS data was not available 10 years prior to dialysis initiation in the current analysis. Although the MDS has been used previously as the “gold standard” for NH care, accuracy has not been reported and is limited only to Medicare or Medicaid certified NHs nor does it include patients living at home who may be receiving equal levels of care through Home Health agencies or paid and informal caregivers. Despite these limitations the large sample size, high PPV, NPV and predictive validity of the CMS-2728 should be considered strengths.

## Conclusion

In conclusion, among incident ESRD patients ≥ 75 years the Medical Evidence Report CMS-2728 NH item identified older adults with functional impairment and increased risk for mortality, however the CMS-2728 may underestimate the burden of NH care by nearly two thirds. Future studies should determine if interventions targeting older adults requiring NH care can improve outcomes, improve care coordination between NHs and dialysis facilities, or support communication with patients about advance care planning.

## Additional file

Additional file 1:
**Accuracy of CMS-2728 using other nursing home definitions.** Outline of the FPAQ questionnaire.

## Abbreviations

NH: Nursing home
CMS: Centers for Medicare & Medicaid Services
ESRD: End-stage renal disease
PPV: Positive predictive value
NPV: Negative predictive value
MDS: Minimum Data Set
CI: Confidence interval
