# Supplementary material for: Underreporting of nursing home utilization on the CMS-2728 in older incident dialysis patients and implications for assessing mortality risk
Source: BMC Nephrol. 2015 Mar 21;16:32. doi: 10.1186/s12882-015-0021-9 (PMC4408561; doi:10.1186/s12882-015-0021-9)
Supplement: Additional file 1: — Accuracy of CMS-2728 using other nursing home definitions. Outline of the FPAQ questionnaire. [file 12882_2015_21_MOESM1_ESM.docx]

| **Supplemental Table. Accuracy of the CMS-2728 nursing home status using multiple definitions for nursing home status (n=27,913).** | | | | |
| --- | --- | --- | --- | --- |
|  | **Estimate (95% CI)** | | | |
|  | **Sensitivity** | **Specificity** | **PPV** | **NPV** |
| Definition 1 | 0.33 (0.32, 0.34) | 0.97 (0.96, 0.97) | 0.80 (0.78, 0.81) | 0.79 (0.78, 0.79) |
| Definition 2 | 0.38 (0.35, 0.41) | 0.90 (0.89, 0.90) | 0.15 (0.14, 0.17) | 0.97 (0.97, 0.97) |
| Definition 3 | 0.38 (0.37, 0.39) | 0.96 (0.95, 0.96) | 0.69 (0.68, 0.71) | 0.86 (0.85, 0.86) |
| Definition 4 | 0.36 (0.35, 0.37) | 0.96 (0.95, 0.96) | 0.75 (0.74, 0.77) | 0.79 (0.79, 0.80) |
| MDS = Minimum Data Set  PPV = positive predictive value  NPV = negative predictive value  **Definition 1.** Patients were defined as requiring nursing home care by the MDS if the last MDS record prior to the ESRD date indicated that they were in a nursing home on the ESRD date or if they had an MDS record indicating nursing home care within 30 days after the ESRD date.  **Definition 2.** Nursing home status for the MDS was defined by an MDS record indicating that the patient was in a NH on the ESRD date (no 30 day window after ESRD date).  **Definition 3.** Nursing home status for the MDS was defined by an MDS record indicating that the patient was in a NH on the CMS-2728 physician signature date (no 30 day window after ESRD date)  **Definition 4.** Nursing home status for the CMS-2728 defined as “1. Assisted living,” “2. Nursing Home,” or “3. Other Institution” on item 17u.Patients were defined as requiring nursing home care by the MDS if the last MDS record prior to the ESRD date indicated that they were in a nursing home on the ESRD date or if they had an MDS record indicating nursing home care within 30 days after the ESRD date. | | | | |
